# Serum NT-proBNP level for predicting functional outcomes after acute ischemic stroke

**DOI:** 10.1038/s41598-023-41233-y

**Published:** 2023-08-25

**Authors:** Phattheera Srisujikul, Kitti Thiankhaw, Surat Tanprawate, Atiwat Soontornpun, Chayasak Wantaneeyawong, Chutithep Teekaput, Nopdanai Sirimaharaj, Angkana Nudsasarn

**Affiliations:** 1 Department of Internal Medicine, Faculty of Medicine, Chiang Mai University, Chiang Mai, Thailand; 2 Division of Neurology, Department of Internal Medicine, Faculty of Medicine, Chiang Mai University, Chiang Mai, Thailand; 3 The Northern Neuroscience Center, Faculty of Medicine, Chiang Mai University, Chiang Mai, Thailand

**Keywords:** Neuroscience, Neurology, Neurological disorders

## Abstract

N-terminus pro-brain natriuretic peptide (NT-proBNP) has been studied and recognized as a biomarker of cardiac thrombogenicity and stroke risk. However, the association between NT-proBNP and functional outcomes following acute ischemic stroke is still debated. This study aimed to investigate whether serum NT-proBNP level is associated with functional outcomes in acute ischemic stroke individuals. This prospective cohort study included patients diagnosed with acute ischemic stroke, and serum NT-proBNP levels were measured within 72 h. At 3 months, all patients were followed up for a modified Rankin Scale (mRS), and logistic regression models were used to evaluate the association of NT-proBNP on the primary outcome, in which a score of 3–6 was classified as an unfavorable functional outcome. Sixty-seven patients were enrolled in the study, and 23 (34.3%) patients were identified with an unfavorable functional outcome. Elevated serum NT-proBNP levels (> 100 pg/mL) were observed in 57 (85.1%) patients, and the Youden index demonstrated a cutpoint estimation of poor outcomes at 476 pg/mL with 74% sensitivity and 63% specificity. Multivariate regression analysis showed an elevation of NT-proBNP above the cutpoint level was an independent predictor for unfavorable functional outcomes, odds ratio 3.77, 95% confidence interval (1.04–13.62), *P* = 0.04. The present study demonstrated that elevated serum NT-proBNP levels were expected among acute ischemic stroke patients and represented the risk of unfavorable functional outcomes, suggesting that NT-proBNP might be a useful biomarker for predicting prognosis after ischemic stroke.

## Introduction

Ischemic stroke is a common neurological disorder that occurs from infarction of the brain parenchyma, spinal cord, or retina, accounting for 80 percent of all stroke cases^1^. Ischemic stroke is a leading cause of morbidity and mortality worldwide, and the functional outcome after an ischemic stroke is essential information that can be used to plan further treatment to improve the patient’s quality of life^2^. Recent studies elucidated the association between cardiac and vascular thrombogenicity biomarkers and ischemic stroke, but biomarkers for predicting functional outcomes following stroke are limited^3–6^. A promising biomarker for predicting functional outcomes from ischemic stroke is N-terminus pro-brain natriuretic peptide (NT-proBNP), secreted predominantly from the heart, lungs, kidneys, adrenal gland, and also the brain^7^.

Previous studies have found that NT-proBNP might be elevated in acute ischemic stroke individuals and demonstrated the association between NT-proBNP levels and various stroke parameters, including cardioembolic stroke and mortality rate after ischemic stroke^8, 9^. Nonetheless, few studies studied the association between NT-proBNP and functional outcomes after ischemic stroke, and the results still lacked a conclusion^10, 11^. The present study aimed to determine the association between NT-proBNP and functional outcomes after ischemic stroke in a prospective cohort.

## Methods

### Study design and participants

A single-center prospective cohort study was conducted at Maharaj Nakorn Chiang Mai Hospital, a University Hospital of the Faculty of Medicine, Chiang Mai University. Acute Ischemic Stroke (AIS) patients admitted to the Comprehensive Stroke Service based at Maharaj Nakorn Chiang Mai Hospital from November 2021 to October 2022 were prospectively enrolled in the study if they were 18 or older and had a stroke onset within 72 h. AIS patients who had stroke symptoms at this time point were initially enrolled in this study because they met the criteria to admit to the acute stroke unit (ASU) and were managed as standard stroke care to minimize treatment effect bias. The sample size estimation is shown in Supplementary Table S1. Acute ischemic stroke was diagnosed by certified board neurologists based on clinical presentation and demonstrated brain infarction on computed tomography (CT) or magnetic resonance imaging (MRI) of the brain. Patients who received treatment with intravenous thrombolysis (IVT), interventional thrombolysis or endovascular treatment, or had a condition that affected serum NT-proBNP levels, including congestive heart failure, acute coronary syndrome, pulmonary diseases (pulmonary embolism, chronic obstructive pulmonary disease), hypertensive urgency or emergency (defined by systolic blood pressure (SBP) ≥ 220 mmHg and/or diastolic blood pressure (DBP) ≥ 120 mmHg), and serum creatinine > 2.5 mg/dL were excluded from the study.

Enrolled patients received treatment in the acute stroke unit by the consultant or vascular neurologist. The standard of care for stroke was applied to the participants, including supplemental oxygen, control and management of blood pressure, temperature, glucose, and intravenous fluid hydration. Antithrombotics, antiplatelet or anticoagulants, were started within 48 h after onset, depending on stroke etiology, unless contraindication has been indicated^12^. The complete list of specific treatment protocols is shown in Supplementary Table S2.

### Blood collection and serum NT-proBNP measurement

At the time of enrollment, 2–4 mL of blood was collected from participants and immediately delivered to the central laboratory unit to measure serum NT-proBNP by the laboratory technicians blinded to the patient's clinical outcomes. We measured NT-ProBNP level by immunoassay method (Elecsys proBNP II), which can measure the level of NT-ProBNP in the range of 5 to 35,000 pg/mL. Before obtaining the cutpoint, serum NT-proBNP levels were categorized into three groups based on reference range and literature review^13^. A level of less than 100 pg/mL was classified as normal, 100–750 pg/mL was identified as an elevated level, and more than 750 pg/mL was recognized as a marked elevation.

### Data collection and outcomes

The data were collected from recruited patients at the enrollment, including demographic characteristics, medical and medication history, laboratory results, classification of subtype of AIS based on the Trial of Org 10172 in Acute Stroke Treatment (TOAST) classification^14^, electrocardiography (EKG) and echocardiography variables, and neuroimaging parameters. The function outcomes of AIS were determined by the National Institutes of Health Stroke Scale (NIHSS), modified Ranking Scale (mRS), and Barthel Index (BI). A primary outcome of the study was the 90-day mRS, which was stratified into seven levels from zero to six. A score of zero indicated no symptoms, while a higher score reflected poorer neurological outcomes. The patients who had mRS scores from 0 to 2 were grouped as favorable functional outcomes, while those with mRS scores of more than two were grouped as unfavorable functional outcomes. We use mRS to evaluate the functional outcome because of its simplified ordinal scale, and the reliability of determining the patient's mRS category score is appropriate regardless of whether it is determined via telemedicine interview or consultation^15^. In addition, numerous categories of evidence support the validity and dependability of the mRS. The reported data support the notion that an mRS is a helpful tool for determining the efficacy of stroke treatments^16^. The secondary outcome was the cutpoint level of serum NT-proBNP that can predict unfavorable functional outcomes at the 90-day follow-up appointment.

### Statistical analysis

The categorical variables were presented with numbers and proportions and used the Pearson χ^2^ test or Fisher exact 2-sided test when appropriate. Mean and corresponding standard deviation (SD), or median and corresponding interquartile range (IQR), were reported for continuous data as appropriate. Comparisons between groups of continuous variables were performed using analysis of variance (ANOVA) or the Kruskal–Wallis test as appropriate.

We evaluated univariable analysis using an odds ratio (OR) with a 95% confidence interval (CI), and variables with statistical significance or P*-*value less than 0.2 or considered as clinical significance were used in multivariable logistic regression analysis to adjust confounding factors. We calculated the cutpoint level of serum NT-proBNP that predicts poor functional outcome at 3 months using Youden index cutpoint estimation and the area under the receiver operating characteristic (AuROC) curve. All statistical analyses were performed using licensed Stata statistical software version 16.1 (Stata Statistical Software: Release 16.1, Stata Corporation, College Station, TX, 2019).

### Ethical approval

This study involved human participants and was approved by The Institutional Review Board of the Faculty of Medicine, Chiang Mai University (Study Code: MED-2564-08312). Participants or their relatives gave informed consent to participate in the study before taking part, and the data were anonymized or maintained with confidentiality. All methods in the study were conducted ethically in accordance with the Declaration of Helsinki.

## Results

### Demographic characteristics

**Figure 1:**
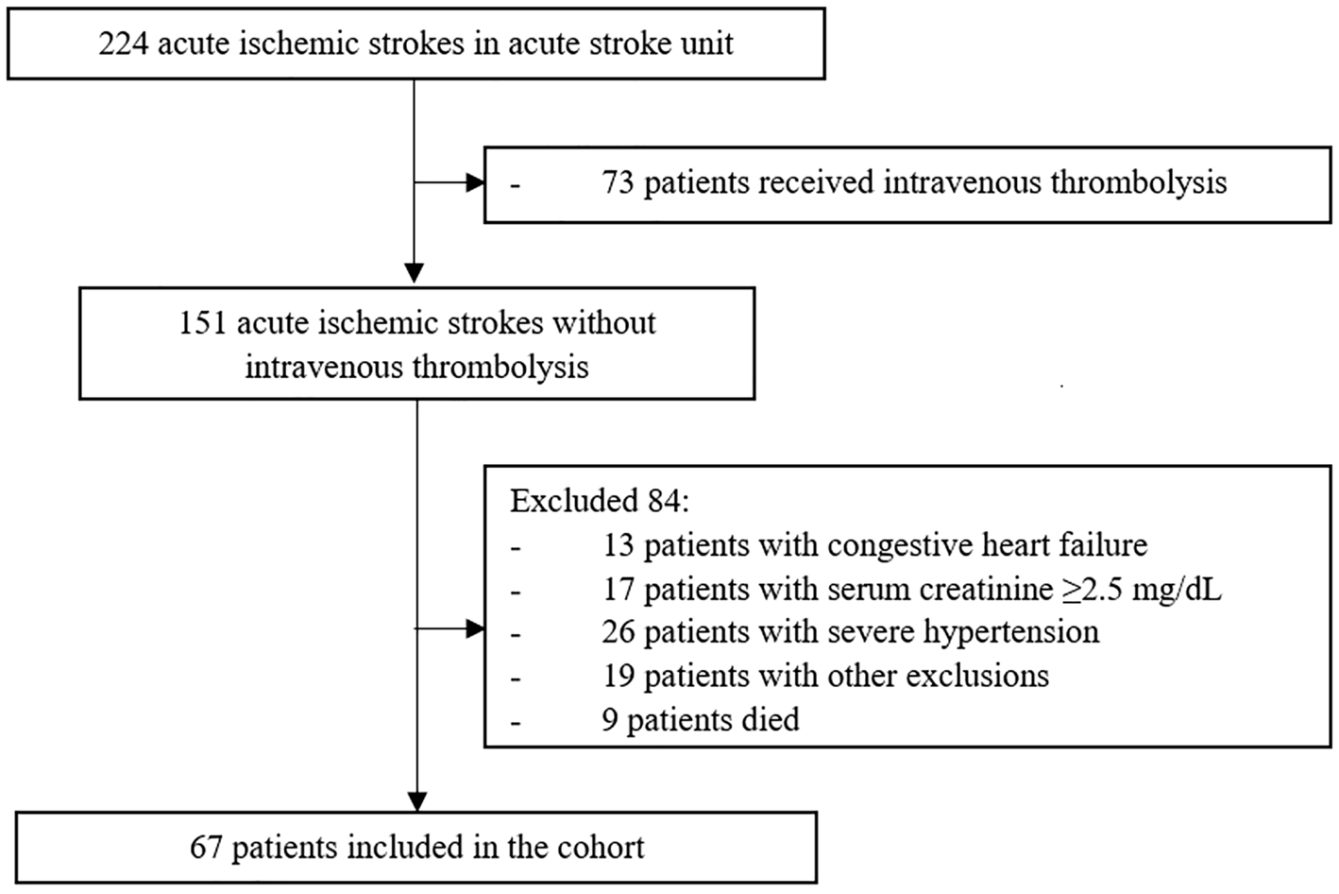
Flow chart of included participants.

Of 224 patients with acute ischemic stroke in the acute stroke unit, 73 patients received IVT and 84 patients were excluded from this study due to at least one of the exclusion criteria. The remaining 67 patients were enrolled in the analyzed cohort (Fig. 1). The baseline characteristics of the participants are presented in Table 1. Men occupied 43.3% (n = 29) of AIS patients, and the mean age of the participants was 66.9 years (± 13.9). Patients with marked elevated serum NT-proBNP levels (> 750 pg/mL) were more elderly (60.3 vs. 62.3 vs. 74.5 years, respectively, P < 0.001), while patients with normal levels had higher body mass index (BMI) (26.5 vs. 22.5 vs. 22.9 km/m^2^, respectively, P = 0.02). Six patients (22.2%) with atrial fibrillation as an underlying disease had serum NT-proBNP of more than 750 pg/mL, significantly higher than patients without atrial fibrillation (P = 0.006). In addition, elevated serum NT-proBNP at 750 pg/mL or more is associated with a higher serum creatinine with a median value of 1.04 mg/dL (IQR 0.8–1.3, P = 0.002), newly diagnosed atrial fibrillation (NDAF) (44.4%, P = 0.002), cardioembolism (51.9%, P = 0.001), left atrial enlargement (LAE) (25.9%, P = 0.03) and enlarged left atrial diameter (mean LADM, 3.9 ± 0.7 cm, P = 0.04). There was no significant difference in other demographics between the groups.

**Table 1:** Baseline characteristics of the patients.

Characteristics	NT-proBNP, pg/mL				
	Total cohort (n = 67)	< 100 (n = 10)	100–750 (n = 30)	> 750 (n = 27)	*P-*value
Demographic					
Age, yr—mean (SD)	66.9 (13.9)	60.3 (10.7)	62.3 (13.6)	74.5 (12.1)	< 0.001
Male sex—no. (%)	29 (43.3)	4 (40.0)	14 (46.7)	11 (40.7)	0.95
BMI, kg/m^2^—mean (SD)	23.2 (3.2)	26.5 (3.8)	22.5 (2.8)	22.9 (3.2)	0.02
Current smoking—no. (%)	16 (27.6)	3 (33.3)	8 (28.6)	5 (23.8)	0.86
Current alcohol drinking—no. (%)	22 (37.9)	3 (33.3)	11 (39.3)	8 (38.1)	1.00
Medical history—no. (%)					
Hypertension	45 (67.2)	7 (70.0)	17 (56.7)	21 (77.8)	0.23
Diabetes mellitus	19 (28.4)	0	10 (33.3)	9 (33.3)	0.10
Dyslipidemia	27 (40.3)	5 (50.0)	9 (30.0)	13 (48.2)	0.30
Atrial fibrillation	6 (9.0)	0	0	6 (22.2)	0.006
Chronic kidney disease	4 (5.97)	0	1 (3.33)	3 (11.11)	0.37
Prior stroke/TIA	10 (14.9)	1 (10.0)	2 (6.7)	7 (25.9)	0.12
Medication history—no. (%)					
Antiplatelet therapy	50 (74.6)	10 (100.0)	27 (90.0)	13 (48.2)	< 0.001
NOAC	12 (17.9)	0	1 (3.3)	11 (40.7)	< 0.001
VKA	4 (5.9)	0	0	4 (14.8)	0.05
Antihypertensive drugs	24 (35.8)	2 (20.0)	8 (26.7)	14 (51.9)	0.08
Lipid-lowering drugs	66 (98.5)	10 (100.0)	30 (100.0)	26 (96.3)	0.55
Clinical features					
Time from stroke onset to serum NT-proBNP, hours—mean (SD)	43.1 (35.2)	50.4 (47.7)	46.2 (39.0)	36.9 (24.4)	0.83
SBP, mmHg—mean (SD)	159.1 (32.3)	157.7 (28.0)	151.5 (34.7)	168.2 (29.7)	0.15
Heart rate, bpm—mean (SD)	78.3 (14.3)	85.7 (13.5)	76.5 (12.0)	77.5 (16.5)	0.20
HDL, mg/dL—median (IQR)	47 (38–57)	42.5 (36–56)	50 (39–58)	46 (39–56)	0.79
LDL, mg/dL—median (IQR)	113 (88–133)	117 (107–136)	127 (96–134)	95 (78–116)	0.11
Fasting blood glucose, mg/dL—median (IQR)	130 (109–162)	131.5 (117–157)	133 (112–179)	129 (100–161)	0.69
Creatinine, mg/dL—median (IQR)	0.87 (0.7–1.1)	0.75 (0.7–0.9)	0.81 (0.6–1.0)	1.04 (0.8–1.3)	0.002
NDAF—no. (%)	15 (22.4)	1 (10.0)	2 (6.7)	12 (44.4)	0.002
TOAST—no. (%)					
LAA	24 (35.8)	6 (60.0)	12 (40.0)	6 (22.2)	0.08
CE	17 (25.4)	0	3 (10.0)	14 (51.9)	0.001
SVO	17 (25.4)	3 (30.0)	9 (30.0)	5 (18.5)	0.41
OD	5 (7.4)	0	3 (10.0)	2 (7.4)	0.38
UD	4 (6.0)	1 (10.0)	3 (10.0)	0	0.25
OCSP—no. (%)					
TACI	1 (1.5)	0	0	1 (3.7)	0.55
PACI	34 (50.7)	4 (40.0)	16 (53.3)	14 (51.9)	0.79
POCI	15 (22.4)	3 (30.0)	6 (20.0)	6 (22.2)	0.91
LACI	15 (22.4)	3 (30.0)	7 (23.3)	5 (18.5)	0.90
Other	2 (3.0)	0	1 (3.3)	1 (3.7)	1.00
ECG and echocardiography					
LAE—no. (%)	8 (11.9)	0	1 (3.3)	7 (25.9)	0.03
LVH—no. (%)	11 (16.4)	1 (10.0)	3 (10.0)	7 (25.9)	0.27
LADM—mean (SD)	3.6 (0.9)	3.2 (0.3)	3.1 (1.0)	3.9 (0.7)	0.04
Neuroimaging					
HMCAS—no. (%)	10 (14.9)	2 (20.0)	3 (10.0)	5 (18.5)	0.58
Anterior ASPECTS—median (IQR)	10 (8–10)	10 (8–10)	10 (8–10)	10 (8–10)	0.99
Posterior ASPECTS—median (IQR)	10 (9–10)	10 (8–10)	10 (10–10)	10 (9–10)	0.43
Large infarction—no. (%)	12 (17.9)	3 (30.0)	3 (10.0)	6 (22.2)	0.24

*ASPECTS* The Alberta Stroke Program Early CT Score, *BMI* body mass index, *CE* cardioembolism, *ECG* electrocardiography, *HDL* high-density lipoprotein, *HMCAS* hyperdense middle cerebral artery sign, *IQR* interquartile range, *LAA* large-artery atherosclerosis, *LACI* lacunar infarct, *LADM* left atrium diameter, *LAE* left atrial enlargement, *LDL* low-density lipoprotein, *LVH* left ventricular hypertrophy, *NDAF* newly diagnosed atrial fibrillation, *NOAC* non-vitamin K antagonist oral anticoagulant, *NT-proBNP* N-terminal fragment B-type natriuretic peptide, *OCSP* the Oxfordshire Community Stroke Project Classification, *OD* other determined etiology, *PACI* partial anterior circulation infarcts, *POCI* posterior circulation infarcts, *SBP* systolic blood pressure, *SD* standard deviation, *SVO* small-vessel occlusion, *TACI* total anterior circulation infarcts, *TIA* transient ischemic attack, *TOAST* the Trial of Org 10172 in Acute Stroke Treatment, *UD* undetermined etiology, *VKA* vitamin K antagonist.

### Functional outcomes and NT-proBNP

**Figure 2:**
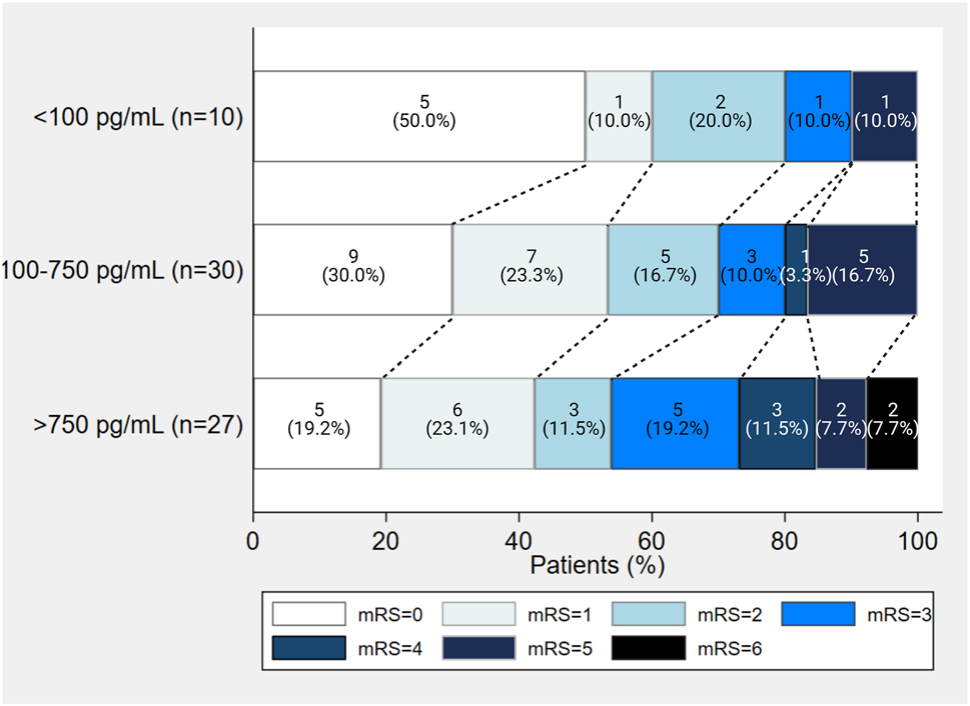
NT-proBNP levels and 90-day functional outcomes of participants.

Table 2 demonstrates the functional outcomes of participants at admission and 90 days according to NT-proBNP levels. There was no statistical difference in clinical outcomes at admission. At 90 days, participants with normal NT-proBNP levels were likely to have better functional outcomes, mRS of 0.5 (0–2) and BI of 97.5 (85–100), although these results were not statistically significant (Fig. 2). Also, the median NIHSS score in admission (5) and 90 days (2) was higher in patients with serum NT-proBNP of more than 750 pg/mL. After empirical cutpoint estimation using the Youden index method, the serum NT-proBNP value of 476 pg/mL predicted unfavorable functional outcomes at 90 days, defined by mRS ≥ 3, with the AuROC curve at cutpoint 0.68, cutpoint sensitivity of 74% and specificity of 63% (Supplementary Table S3).

**Table 2:** NT-proBNP levels and functional outcomes of participants at admission and 90 days.

Functional outcomes	NT-proBNP, pg/mL			
	< 100 (n = 10)	100–750 (n = 30)	> 750 (n = 27)	*P*-value
Admission—median (IQR)				
NIHSS score	2.5 (2–4)	3 (2–5)	5 (3–9)	0.10
mRS	3 (2–4)	3 (2–4)	3.5 (3–4)	0.08
BI	67.5 (50–70)	62.5 (35–75)	37.5 (30–70)	0.14
90-day—median (IQR)				
NIHSS score	1 (0–2)	1 (0.5–4)	2 (1–4)	0.40
mRS	0.5 (0–2)	1 (0–3)	2 (1–4)	0.23
BI	97.5 (85–100)	90 (65–100)	80 (50–100)	0.20

*BI* Barthel Index, *IQR* interquartile range, *mRS* modified Rankin Scale, *NIHSS* National Institute of Health Stroke Scale, *NT-proBNP* N-terminal fragment B-type natriuretic peptide.

### NT-proBNP cutpoint

**Figure 3:**
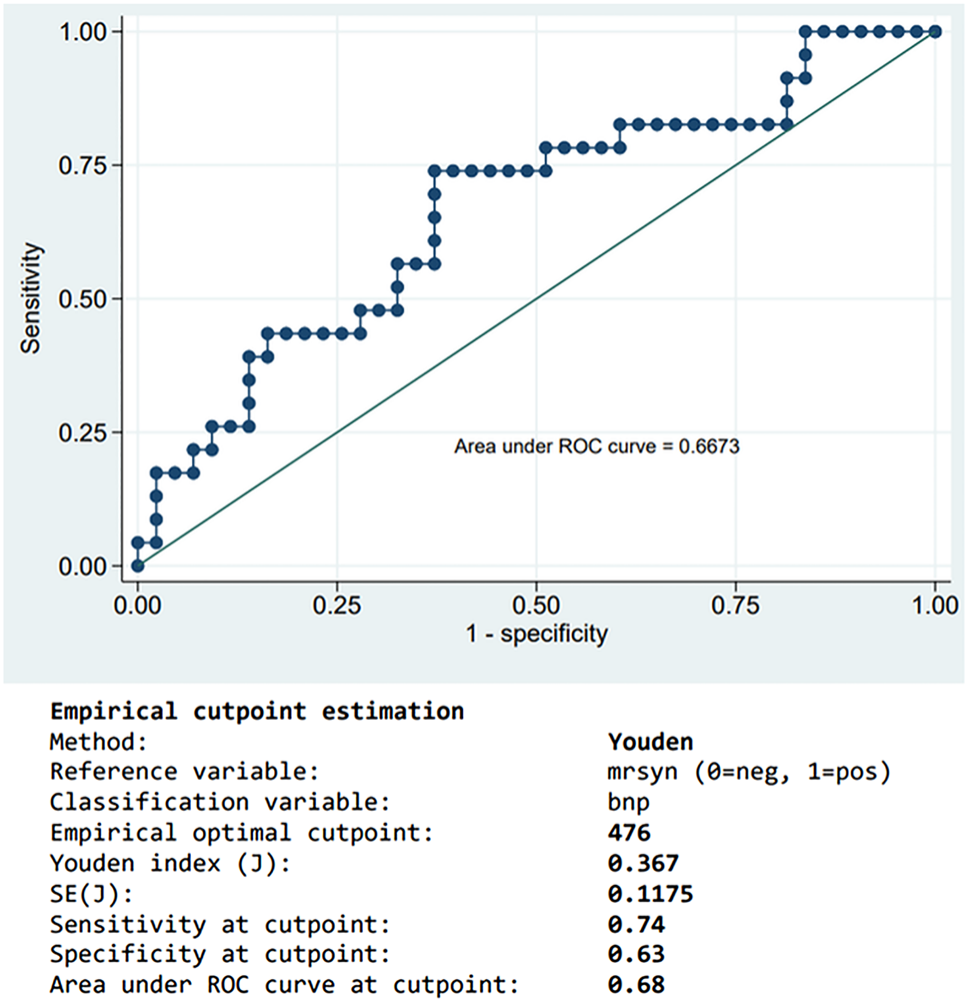
Receiver operating characteristic (ROC) curve of NT-proBNP cutpoint levels for predicting an unfavorable functional outcome (mRS ≥ 3).

The univariable analysis is shown in Supplementary Table S4 and multivariable models are shown in Table 3 and Supplementary Table S5. The cutpoint value of serum NT-proBNP at 476 pg/mL was used to evaluate the association with unfavorable functional outcomes at 90 days and divided patients into two groups, as shown in Table 3 and Supplementary Fig. S1. Seventeen patients (51.5%) with serum NT-proBNP 476 pg/mL or more had an unfavorable functional outcome, while 18.2% of patients with serum NT-proBNP less than the cutpoint value had unfavorable functional outcomes (Fig. 3). Serum NT-proBNP at 476 pg/mL or more was associated with unfavorable functional outcomes in the unadjusted model (unadjusted OR 4.80; 95% CI, 1.56–14.62; P = 0.006). As demonstrated in Table 3, participants with a higher serum NT-proBNP level were independently associated with a risk of unfavorable functional outcomes after being adjusted with confounding factors, including age, serum creatinine, atrial fibrillation, and large infarction.

**Table 3:** 90-day unfavorable functional outcomes at cutpoint of NT-proBNP levels at 476 pg/mL among acute ischemic stroke patients.

	NT-proBNP, pg/mL		
	< 476 (*n* = 33)	≥ 476 (*n* = 33)	*P-*value
Unfavorable functional outcome: mRS ≥ 3			
Events—No. (%)	6 (18.2)	17 (51.5)	
OR (95% CI)			
Unadjusted	1	4.80 (1.56–14.62)	0.006
Multivariable model 1	1	5.86 (1.48–23.27)	0.01
Multivariable model 2	1	3.77 (1.04–13.62)	0.04
Multivariable model 3	1	4.81 (1.34–17.26)	0.02
Multivariable model 4	1	4.00 (1.10–13.88)	0.04
Multivariable model 5	1	5.03 (1.43–17.72)	0.01

*CI* confident interval, *mRS* modified Rankin Scale, *NT-proBNP* N-terminal fragment B-type natriuretic peptide, *OR* Odds ratio.

Multivariable model 1: age, sex, time from stroke onset to serum NT-proBNP.

Multivariable model 2: age, atrial fibrillation, prior stroke, or transient ischemic attack.

Multivariable model 3: antihypertensive drugs, serum creatinine, prior stroke or transient ischemic attack.

Multivariable model 4: antihypertensive drugs, hyperdense middle cerebral artery sign, large infarction.

Multivariable model 5: atrial fibrillation, hyperdense middle cerebral artery sign, large infarction.

## Discussion

The study aimed to document the association between NT-proBNP and functional outcomes in acute ischemic stroke patients. Our findings found that elevated serum NT-proBNP levels commonly occurred following acute ischemic stroke, following the previous studies, which was illustrated in 44–65.5% of acute stroke patients^10, 11^. The present study showed that elevated serum NT-proBNP level in the acute phase of stroke was significantly associated with an increased risk of unfavorable functional outcomes. In addition, our study found the cutpoint estimation value of NT-ptoBNP at 476 pg/mL as an independent predictor for stroke outcomes after being adjusted with potential confounding factors.

Several studies were conducted to elucidate the potential use of serum NT-proBNP as a biomarker for predicting ischemic stroke outcomes^17, 18^. An analysis of ischemic stroke patients with elevated blood pressure from the China Antihypertensive Trial in Acute Ischemic Stroke (CATIS) showed that raised NT-proBNP levels increased the risk of poor functional outcomes and other composite endpoints, including mortality and vascular events at 1 year^18^. Similarly, the findings by Mäkikallio et al. found that plasma brain natriuretic peptides (N-BNP) remained an independent predictor of poststroke mortality, a risk ratio of 3.7 95% CI 1.4–10.0, P < 0.05^19^. These findings were supported by a prospective study by Chaudhuri et al*.*, which found the association of plasma N-BNP elevation as a biomarker for poor stroke outcomes at the 90-day consecutive follow-up, and also for cardioembolism stroke classification^11^. However, some contradictory findings still exist in a study on 174 acute ischemic stroke patients with MRI-confirmed ischemic stroke without evident myocardial damage. Blood biomarkers, cardiac troponin T (cTnT), and NT-proBNP did not significantly relate to clinical outcomes. The factors influencing treatment outcomes still relied on stroke severity, infarct size, and patient age^10^. A systematic review and meta-analysis of 4523 AIS patients demonstrated that patients with elevated levels of NT-proBNP were associated with unfavorable functional outcomes (OR 1.68) with high heterogeneity (I^2^ = 90.8%)^9^, which can be implied that more studies are needed to establish the association of NT-proBNP with functional outcome after ischemic stroke.

Mechanisms for the association of stroke outcomes and NT-proBNP are uncertain and need further exploration. It may have resulted from local hypoxia and ischemic injury of brain tissues, inappropriate release of nerve transmitters triggered by intense stimulation of the hypothalamo-hypophyseal system, or strain of blood vessel wall caused by hemodynamic alterations^20, 21^. Consequently, the effects from different ischemic locations, especially the thalamus and hypothalamus, may influence NT-proBNP levels and have implications in clinical practice. Ventricular dysfunction and autonomic dysfunction or sympathetic overactivation might occur following acute neuronal stress or injury, in this setting is acute ischemic stroke, and NT-proBNP is released in response to these conditions^22, 23^. Although NT-proBNP level might predict functional outcomes in ischemic stroke individuals, considering these factors and other important predictors should be considered.

In the previous studies for NT-proBNP cutpoint in predicting clinical outcomes of stroke, stroke patients with elevated NT-proBNP levels of more than 77 to 100 pg/mL were observed in approximately three-fourths of patients and correlated with poor functional outcomes^11, 24^. On the other hand, our study demonstrated that serum NT-proBNP levels of 476 pg/mL might be used as a potential cutpoint for clinical outcomes of stroke. Our cutpoint value was substantially higher than the previous studies but could be considered for practical use as a screening parameter due to its acceptable sensitivity. Further studies should be performed to establish the optimal cutpoint value, especially for its application in routine clinical practice.

Cardioembolism and even newly-diagnosed atrial fibrillation were considerably documented in participants with elevated serum NT-proBNP levels, which is more evident in marked elevation groups (> 750 pg/mL). These findings are concordant with a previous report, which demonstrated that high levels of NT-proBNP might predict the development of atrial fibrillation with cerebral infarction. In a prospective study of patients with cryptogenic stroke, pro-BNP ≥ 360 pg/mL increases the likelihood of AF detection during follow-up by a factor of five^25^. Although previous studies have shown that NT-proBNP levels might have started to decline 48 h after onset, a pooled data meta-analysis supported these findings that BNP/NT-proBNP levels were noticeably raised in cardioembolic stroke up to 72 h after the stroke onset^8^ and may remain elevated at day 6 in acute ischemic stroke^26^. Several prospective studies have been conducted to demonstrate the predictive value of NT-proBNP after ischemic stroke, including all-cause or cardiovascular mortality and functional outcomes, usually collected blood between 4 and 72 h after hospital admission^9^. In addition, the rising serum NT-proBNP levels in the acute setting of ischemic stroke could be a surrogate biomarker for atrial cardiopathy, as observed for left atrial enlargement and increased left atrial diameter in our cohort. According to an analysis from the Warfarin–Aspirin Recurrent Stroke Study (WARSS), a subpopulation of ischemic stroke patients with elevated NT-proBNP concentrations of more than 750 pg/mL may benefit more from anticoagulants than antiplatelet medications^13^. Interestingly, a retrospective study of patients with embolic stroke of undetermined source (ESUS) found that NT-proBNP > 250 pg/mL is associated with an increased risk of death in this population^27^. These findings emphasized the predictive value of NT-proBNP after ischemic stroke and its utilization with various stroke parameters, especially atrial cardiopathy, and subtype of stroke for antithrombotic guidance.

Apart from NT-proBNP levels, our findings found that some factors have also affected functional outcomes following acute ischemic stroke, including age, ischemic stroke subtypes, antithrombotics, and BMI or obesity. Several studies established the influence of age, atrial fibrillation, and anticoagulants used on the disability and mortality of ischemic stroke patients^28, 29^. BMI or obesity is an increased study to clarify the association of BMI with mortality and functional outcomes after ischemic stroke. Being overweight or obese was associated with lower mortality or improved functional recovery in ischemic stroke patients, especially in cardioembolism and small-vessel occlusion subtypes, whereas being underweight predicted poor outcomes^30, 31^. This effect is known as the obesity paradox and may be explained through the potential protecting effects of adipose tissue^32^.

The strength of this current study was that a prospective study in which well-controlled potential factors might be affected the value of serum NT-proBNP, including time from symptoms onset and other comorbidities. Our results provide scientific evidence to support other previous studies regarding the possibility of blood biomarkers for predicting functional outcomes of stroke. Moreover, this hospital-based study proposed a cutpoint level of NT-proBNP and showed elevated levels in acute ischemic stroke with cardioembolism or markers of left atrial cardiopathy, including left atrial enlargement and increased left atrial diameter.

However, we acknowledge some limitations to our study. First, because of a single-center study in the Thai population, the results and conclusion may not implement across all ischemic stroke populations. Second, we initially planned the serial measurement of serum NT-proBNP levels to determine the rise and fall patterns and different values of NT-proBNP, which is probably a more accurate prediction. Because of the lack of funding, we amended the study protocol to perform a single initial value at enrollment instead. Further study in other populations and two-time points measurement is encouraged to close this knowledge gap. Finally, patients with AIS enrolled in this study appear to have mild or moderate stroke severity because individuals with more severity usually met exclusion criteria (e.g., IVT or endovascular treatment) to control confounding of the functional outcomes. Thus, the practicality of NT-proBNP as an indicator of prediction for functional outcomes in patients with severe stroke remains uncertain.

## Conclusions

In acute ischemic stroke patients, elevated serum NT-proBNP levels were commonly found and independently associated with unfavorable functional outcomes at 3 months, especially those markedly elevated or above the empirical optimal cutpoint. Furthermore, greater NT-proBNP serum concentration was associated with a certain subtype of ischemic stroke, newly-diagnosed atrial fibrillation, cardioembolism, and left atrial enlargement. As a result, serum NT-proBNP levels in acute settings of stroke could be used as a reliable marker for ischemic stroke prognostication.

### Supplementary Information

Supplementary Information.

## Data Availability

The study data are available from the corresponding author upon reasonable request and with the permission of all contributing authors.
