# Supplementary material for: Serum NT-proBNP level for predicting functional outcomes after acute ischemic stroke
Source: Sci Rep. 2023 Aug 25;13:13903. doi: 10.1038/s41598-023-41233-y (PMC10457328; doi:10.1038/s41598-023-41233-y)
Supplement: Supplementary file 1 — Supplementary Information. [file 41598_2023_41233_MOESM1_ESM.docx]

**Supplementary Material**

**Table S1.** The sample size estimation.

| **Method** | Estimating two-sample proportions; Pearson's chi-squared test   - Null hypothesis *p_2_* = *p*_1_ and - Alternative hypothesis *p_2_* ≠ *p_1_* (two-sided test), *p_2_* > *p_1_* or *p_2_* < *p_1_* (one-sided test) |
| --- | --- |
| **Imputed** | *α* = significance level = 0.0500  *β* = probability of type II error  Power = 0.8000  $n_{1}= m$ **=** estimated sample size in group 1  $n_{2}=rm$ **=** estimated sample size in group 2  $r=ratio of sample size$  N2/N1 = 4.0000  $P_{1}$= probability of success in group 1 = 0.1556  $P_{2}$= probability of success in group 2 = 0.6852; delta = 0.5296  $\bar{P}= \frac{\left( P_{1}+ {rP}_{2} \right)}{(r+1)}$  $\bar{Q}=1- \bar{P}$ |
| **Required sample size** | $m= \frac{m^{'}}{4}\left( 1+ \sqrt{1+ \frac{2(r+1)}{m^{'}r\left\vert P_{2}- P_{1} \right\vert}} \right)^{2}$ |
| **Result** | N1 = 12  N2 = 48  N = 60 |
| **References** | 1. STATA Power and Sample Size Reference Manual, StataCorp. 2015. Stata: Release 14. Statistical Software. College Station, TX: StataCorp LP. 2. Fleiss, J. L., B. Levin, and M. C. Paik. 2003. Statistical Methods for Rates and Proportions. 3rd ed. New York: Wiley. page 75 -77 |

**Table S2.** The complete list of specific treatment protocols.

| **All participants** | **Standard of care** | **Details** | | |
| --- | --- | --- | --- | --- |
|  | Stroke units | AIS patients were admitted to the acute stroke unit for comprehensive specialized stroke care and incorporated rehabilitation. | | |
|  | Airway, breathing, oxygenation | - Airway support and ventilatory assistance are given in AIS patients with decreased consciousness or bulbar dysfunction that causes compromise of the airway. - Supplemental oxygen is provided to maintain an oxygenation saturation of >94%. | | |
|  | Blood pressure | No early treatment of hypertension unless the following indications have been indicated   - Intravenous thrombolysis candidates with BP >180/105 mmHg - Acute coronary event, acute heart failure, acute aortic dissection, postfibrinolysis symptomatic intracerebral hemorrhage (sICH) - SBP >220 mmHg and/or DBP 121-140 mmHg - DBP >140 mmHg | | |
|  | Temperature | Identified and treatment of hyperthermia (BT >38 ^o^C) | | |
|  | Blood glucose | - Treatment of hypoglycemia when blood glucose <60 mg/dL - Treatment of hyperglycemia to maintain blood glucose in a range of 140-180 mg/dL | | |
|  | IV fluid | Isotonic solution (0.9% NaCl) is recommended in AIS patients within 72 hours and maintains euvolemic status. | | |
|  | Antiplatelet treatment | Antiplatelet agents are administered in noncardioembolic AIS. | | |
|  | Anticoagulants | Anticoagulants (vitamin K antagonist: VKA or direct oral anticoagulants: DOAC) are recommended in cardioembolic AIS. | | |
|  | Treatment of hyperlipidemia | Lipid-lowering therapy with high-intensity statin therapy (atorvastatin 40-80 mg/day) is started in most AIS patients. | | |
|  | Rehabilitation | Early rehabilitation is recommended in AIS without contraindications. | | |
|  | DVT prophylaxis | Intermittent pneumatic compression (IPC) was applied in immobile stroke patients without contraindications. | | |
| **Treatment according to TOAST classification** | | | | |
| LAA | CE | SVO | OD | UD |
| - Aspirin 160-300 mg/day within 48 hours after onset - DAPT: clopidogrel 300 mg loading dose, 75 mg/day + aspirin 81 mg/day x 21 days in TIA high-risk (ABCD_2_ ≥4) or minor nonce stroke within 24 hours after onset | - VKA, INR 2-3 (2.5-3.5 in mechanical heart valves) - DOACs, unless AF with mechanical heart valves or moderate-severe mitral stenosis | Antiplatelet agents | According to the cause identified   - Steroids (vasculitis) - Anticoagulants (extracranial arterial dissection) | Antiplatelet agents |

**Abbreviations:** AF, atrial fibrillation; AIS, acute ischemic stroke; BT, body temperature; CE, cardioembolism; DAPT, dual antiplatelet therapy; DBP, diastolic blood pressure; DVT, deep vein thrombosis; INR, international normalized ratio; IV, intravenous; LAA, large-artery atherosclerosis; OD, stroke of other determined etiology; SBP, systolic blood pressure; SVO, small-vessel occlusion; TIA, transient ischemic attack; UD, stroke of undetermined etiology.

**Table S3.** Cutpoint of NT-proBNP with sensitivity and specificity.

|  | **NT-proBNP (pg/mL)** | **AUC at a cutpoint** | **Sensitivity** | **Specificity** |
| --- | --- | --- | --- | --- |
| Cutpoint | 476 | 0.68 | 74% | 63% |

**Abbreviations:** AUC, area under the curve; NT-proBNP, N-terminal fragment B-type natriuretic peptide.

**Table S4.** Univariable analysis.

| **Characteristics** | **OR** | **95% CI** | ***P-*value** |
| --- | --- | --- | --- |
| NT-proBNP, pg/mL |  |  |  |
| <476 (n=33) | 1.00 | Reference | Reference |
| ≥476 (n=33) | 4.80 | 1.56-14.62 | 0.006 |
| Age | 1.03 | 0.10-1.80 | 0.09 |
| Male sex | 1.23 | 0.44-3.45 | 0.69 |
| Smoking | 1.12 | 0.32-3.92 | 0.87 |
| Alcohol | 0.55 | 0.16-1.86 | 0.34 |
| Hypertension | 2.35 | 0.73-7.54 | 0.15 |
| Diabetes mellitus | 0.91 | 0.30-2.86 | 0.87 |
| Dyslipidemia | 0.74 | 0.26-2.12 | 0.58 |
| Atrial fibrillation | 2.00 | 0.37-10.82 | 0.42 |
| Prior stroke/TIA | 2.11 | 0.54-8.23 | 0.28 |
| Chronic kidney disease | 0.25 | 0.01-5.00 | 0.36 |
| Antiplatelet therapy | 0.50 | 0.16-1.54 | 0.22 |
| Lipid-lowering drugs | 0.20 | 0.01-4.41 | 0.30 |
| Antihypertensive drugs | 2.82 | 1.00-8.10 | 0.05 |
| SBP | 1.01 | 1.00-1.24 | 0.34 |
| Heart rate | 1.00 | 1.00-1.03 | 0.83 |
| Time from stroke onset to serum NT-proBNP | 1.00 | 1.00-1.01 | 0.63 |
| Creatinine | 1.00 | 0.30-3.42 | 1.00 |
| HDL | 1.00 | 0.95-1.01 | 0.28 |
| LDL | 1.00 | 1.00-1.02 | 0.16 |
| TOAST | 0.63 | 0.39-1.03 | 0.07 |
| OSCP | 0.30 | 0.12-0.64 | 0.003 |
| Admission NIHSS score | 2.04 | 1.40-3.00 | <0.001 |
| Admission mRS | 30.0 | 6.50-133.30 | <0.001 |
| LAE | 2.05 | 0.50-9.11 | 0.34 |
| LVH | 1.10 | 0.30-4.20 | 1.00 |
| LADM | 1.50 | 0.61-3.61 | 0.38 |
| HMCAS | 5.83 | 1.34-25.41 | 0.02 |
| Anterior ASPECTS | 0.60 | 0.42-0.84 | 0.003 |
| Posterior ASPECTS | 1.40 | 0.71-2.65 | 0.35 |
| Large infarction | 5.20 | 1.36-19.90 | 0.02 |
| NDAF | 2.74 | 0.84-8.92 | 0.09 |

**Abbreviations:** ASPECTS, The Alberta Stroke Program Early CT Score; BMI, body mass index; CE, cardioembolism; CI, confidence interval; HDL, high-density lipoprotein; HMCAS, hyperdense middle cerebral artery sign; LADM, left atrium diameter; LAE, left atrial enlargement; LDL, low-density lipoprotein; LVH, left ventricular hypertrophy; NDAF, newly diagnosed atrial fibrillation; NT-proBNP, N-terminal fragment B-type natriuretic peptide; OCSP, the Oxfordshire Community Stroke Project Classification; OR, Odds ratio; SBP, systolic blood pressure; TIA, transient ischemic attack; TOAST, the Trial of Org 10172 in Acute Stroke Treatment.

**Table S5.** 90-day functional outcomes according to NT-proBNP levels among acute ischemic stroke patients.

|  | **NT-proBNP, pg/mL** | | | |
| --- | --- | --- | --- | --- |
|  | **<100**  **(n=10)** | **100-750**  **(n=30)** | **>750**  **(n=27)** | ***P-*value** |
| Unfavorable functional outcome: mRS ≥3 |  |  |  |  |
| Events – No. (%) | 2 (20.0) | 9 (30.0) | 12 (44.4) |  |
| OR (95% CI) |  |  |  |  |
| Unadjusted | 1 | 1.71 (0.30-9.72) | 3.43 (0.61-19.35) | 0.54 |
| Multivariable model 1 | 1 | 1.68 (0.28-9.87) | 2.68 (0.41-17.57) | 0.57 |
| Multivariable model 2 | 1 | 2.02 (0.32-12.74) | 1.72 (0.19-15.65) | 0.46 |
| Multivariable model 3 | 1 | 1.36 (0.13-14.29) | 1.49 (0.09-25.01) | 0.78 |

**Abbreviations:** CI, confident interval; mRS, modified Rankin Scale; NT-proBNP, N-terminal fragment B-type natriuretic peptide; OR, Odds ratio.

Multivariable model 1: adjusted for age, sex, and body mass index.

Multivariable model 2: model 1 plus serum creatinine, atrial fibrillation, newly diagnosed atrial fibrillation, prior stroke or transient ischemic attack, and left atrial enlargement.

Multivariable model 3: model 2 plus Oxfordshire Community Stroke Project Classification (partial anterior circulation infarcts).


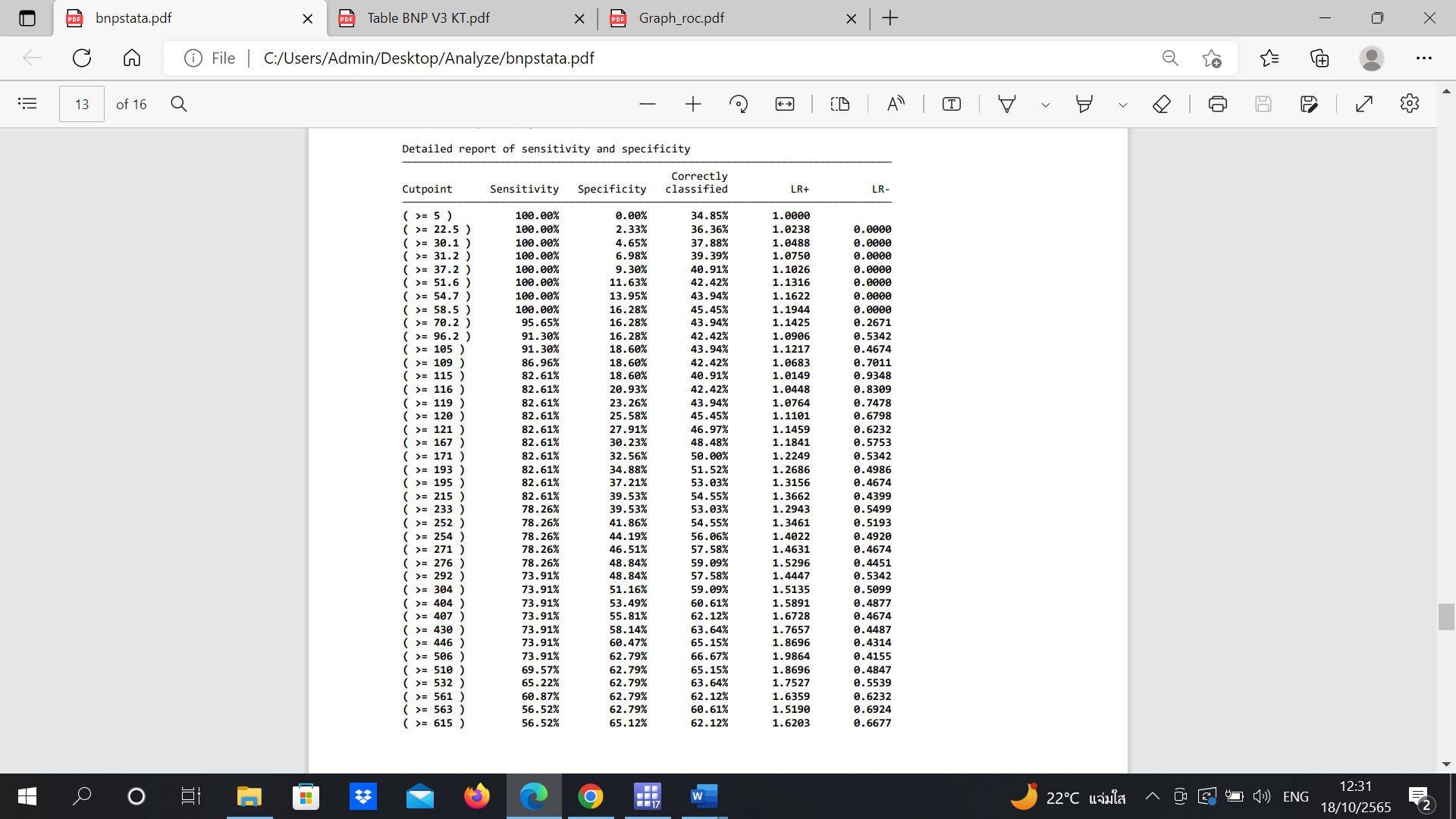
**Figure S1.** Sensitivity, specificity, and likelihood ratio for a positive and negative test of each NT-proBNP cutpoint.
